# GLUT4 in Mouse Endometrial Epithelium: Roles in Embryonic Development and Implantation

**DOI:** 10.3389/fphys.2021.674924

**Published:** 2021-06-25

**Authors:** Yun Long, Yi-cheng Wang, Dong-zhi Yuan, Xin-hua Dai, Lin-chuan Liao, Xue-qin Zhang, Li-xue Zhang, Yong-dan Ma, Yi Lei, Zhi-hui Cui, Jin-hu Zhang, Li Nie, Li-min Yue

**Affiliations:** ^1^Department of Physiology, West China School of Basic Medical Sciences and Forensic Medicine, Sichuan University, Chengdu, China; ^2^Department of Physiology, Chongqing Three Gorges Medical College, Chongqing, China; ^3^West China School of Basic Medical Sciences and Forensic Medicine, Sichuan University, Chengdu, China

**Keywords:** glucose transporter 4, endometrial epithelium, uterine receptivity, embryonic development, implantation

## Abstract

GLUT4 is involved in rapid glucose uptake among various kinds of cells to contribute to glucose homeostasis. Prior data have reported that aberrant glucose metabolism by GLUT4 dysfunction in the uterus could be responsible for infertility and increased miscarriage. However, the expression and precise functions of GLUT4 in the endometrium under physiological conditions remain unknown or controversial. In this study, we observed that GLUT4 exhibits a spatiotemporal expression in mouse uterus on pregnant days 1–4; its expression especially increased on pregnant day 4 during the window of implantation. We also determined that estrogen, in conjunction with progesterone, promotes the expression of GLUT4 in the endometrial epithelium *in vivo* or *in vitro*. GLUT4 is an important transporter that mediates glucose transport in endometrial epithelial cells (EECs) *in vitro* or *in vivo*. *In vitro*, glucose uptake decreased in mouse EECs when the cells were treated with GLUT4 small interfering RNA (siRNA). *In vivo*, the injection of GLUT4-siRNA into one side of the mouse uterine horns resulted in an increased glucose concentration in the uterine fluid on pregnant day 4, although it was still lower than in blood, and impaired endometrial receptivity by inhibiting pinopode formation and the expressions of leukemia inhibitory factor (LIF) and integrin ανβ3, finally affecting embryonic development and implantation. Overall, the obtained results indicate that GLUT4 in the endometrial epithelium affects embryo development by altering glucose concentration in the uterine fluid. It can also affect implantation by impairing endometrial receptivity due to dysfunction of GLUT4.

## Introduction

Embryo implantation is a complex process in which the developing blastocyst adheres to and embeds into the receptive endometrium (Zhang et al., 2013; Kim and Kim, 2017), and these two events are precisely regulated by maternal hormones, in particular ovarian estrogen and progesterone (Cha et al., 2012; Kim and Kim, 2017). In this process, the uterine epithelium, especially the luminal epithelial (LE) cells, is the first site of contact between the maternal and fetal tissues and serves as the transient gateway for embryo development and subsequent implantation in the uterus, and this tissue must undergo sequential specialized changes for successful implantation (Chan et al., 2012; Xie et al., 2018; Ye, 2020). Therefore, any functional defects of the endometrial epithelium may directly or indirectly affect embryo recognition, adhesion, and subsequent implantation. Glucose, one of the important nutrients in uterine fluids and an energy source, is extremely vital for early embryonic development and the endometrial functional activities related to implantation. Some studies have revealed that the embryo uses glucose as its preferred energy source once it enters the uterine cavity (Harris et al., 2005; Gao et al., 2009; Salamonsen et al., 2016). Moreover, adequate glucose uptake and metabolism is essential for the proper differentiation of the endometrium into a receptive state that supports embryo implantation (Frolova and Moley, 2011b; Zhang et al., 2020). Progressively accumulating evidences suggest that glucose metabolism plays important roles in embryonic development, implantation, and pregnancy (Korgun et al., 2001; von Wolff et al., 2003; Doblado and Moley, 2007; Schmidt et al., 2009; Frolova and Moley, 2011a; McKinnon et al., 2014; Zhang et al., 2020). However, glucose uptake and utilization in the endometrial epithelium and its transportation between the uterine fluid and maternal blood circulation remain elusive.

Facilitative glucose transporter molecules, also known as GLUTs, are from the SLC2A family with 14 subtypes responsible for glucose transport across cellular membranes. Studies have revealed that the distribution and function of some types of GLUTs in organisms have tissue and cell specificity (Augustin, 2010; Frolova and Moley, 2011a). Different GLUTs localizing in various cells show different affinities for glucose, which adapt to their specific metabolic demand (von Wolff et al., 2003; Scheepers et al., 2004; Krzeslak et al., 2012). So far, some types of GLUTs have been detected in the uterus of mouse (Frolova and Moley, 2011a), rat (Korgun et al., 2001), ovine (Gao et al., 2009), and human (von Wolff et al., 2003; McKinnon et al., 2014). For example, GLUT1 was reported to be the most abundant GLUT in the endometrium and remained at a high level in early pregnancy. Women with idiopathic infertility had significantly lower levels of GLUT1 expression in the endometrial stromal cells (von Wolff et al., 2003). GLUT3 was reported to be present in tissues that have a high metabolic demand, such as the decidua and the trophoblast (Simpson et al., 2008), while GLUT3 mutations caused early pregnancy loss (Ganguly et al., 2007). A recent study revealed that GLUT6 is upregulated in human endometrial cancer and that inhibiting GLUT6 expression with small interfering RNA (siRNA) reduced the glucose uptake and glycolysis and killed human endometrial cancer cells (Byrne et al., 2014). Moreover, existing data suggested that GLUT dysfunction is closely associated with infertility or endometrial cancer (Wang et al., 2000; Goldman et al., 2006). But little is known about the expression and the functional significance of these GLUTs in the uterine endometrium under normal physiological conditions.

Among them, GLUT4 is likely the most studied GLUT isoform due to the major roles it plays in whole-body glucose homeostasis and in the pathogenesis of type II diabetes mellitus (Klip et al., 2019). Aberrant glucose metabolism by GLUT4 dysfunction in the endometrium could be responsible for the increased miscarriage rates in women with type 2 diabetes or polycystic ovary syndrome (PCOS) (Zhai et al., 2012; Schulte et al., 2015; Alam et al., 2016). GLUT4 is an insulin-dependent glucose transporter that regulates rapid glucose uptake, which contributes to glucose homeostasis under physiological and pathological conditions in skeletal (Deshmukh et al., 2015) and cardiac (Wilson et al., 2013) muscle, adipocytes (Kahn, 2019), and placental cells (Stanirowski et al., 2017); however, its expression and precise functions in the endometrium remain unknown or controversial. Some studies have demonstrated the presence of GLUT4 messenger RNA (mRNA) and protein in human and rodent uterine tissues, and the expression of GLUT4 appears to be regulated in a menstrual cycle-dependent manner (Frolova and Moley, 2011a; Cabrera-Cruz et al., 2020; Neff et al., 2020), whereas some studies have indicated that GLUT4 mRNA and protein are undetectable in human uterus (von Wolff et al., 2003; McKinnon et al., 2014; Cui et al., 2015). In a word, existing studies have suggested the importance of investigating the functions of GLUT4 under physiological conditions, which have sparked our interest in GLUT4 expression and its possible roles in embryonic development and implantation.

In this study, taking mouse as the model, we investigated the expression of GLUT4 in mouse endometrium on pregnant days 1–4 and identified the regulatory effects of ovarian hormones [estrogen (E2) and progesterone (P4)] on GLUT4 using *in vivo* and *in vitro* models. We demonstrated that GLUT4 is an important transporter that mediates the glucose uptake and transport in mouse endometrial epithelial cells (EECs). In addition, the concentration of glucose in the uterine fluid on pregnant day 4 was significantly increased after GLUT4-siRNA was injected into the uterine horn, which resulted in impaired embryo development. The embryo implantation rate was also significantly reduced when GLUT4-siRNA was injected into the uterine horn due to impaired endometrial receptivity through inhibition of the expressions of pinopodes, leukemia inhibitory factor (LIF), and integrin ανβ3. This is true even if excellent embryos were selected for transfer. We highly suspect that GLUT4, which participates in epithelium glucose uptake and transport, not only contributes substantially to the glucose microenvironment in the uterine fluid but is also important for adequate glucose uptake of cells of the uterus *per se* to reach the state of uterine receptivity, therefore interfering with subsequent implantation. Here, we propose a possible mechanism to explain how implantation is affected by the endometrial GLUT4 under physiological conditions. Hopefully, our work will provide a little help in explaining how infertility is affected by dysfunction of GLUT4 in the endometrium.

## Results

### GLUT4 Exhibits Spatiotemporal Expression in the Mouse Endometrium

**FIGURE 1:**
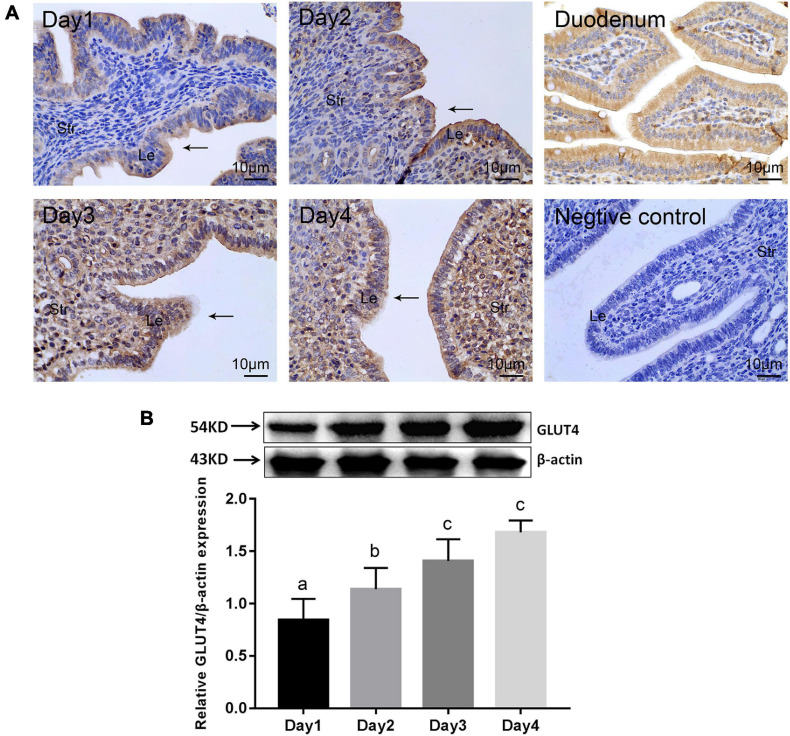
| GLUT4 expression in the mouse endometrium on pregnant days 1–4. Mouse uterine tissues were collected on days 1–4 of pregnancy. One side of the uterine horns was fixed in 4% paraformaldehyde for paraffin blocks and the other side was stored at −80°C for Western blotting analysis, *n* = 10 per group. **(A)** Immunohistochemistry (IHC) staining for GLUT4 expression in the mouse endometrium. *Le*, luminal epithelium; *Str*, stromal tissue. *Arrows* indicate the luminal epithelium. GLUT4 expression in the mouse duodenum for the positive control, without the primary antibody for the negative control. *Scale bar*, 10 μm. **(B)** Western blotting analysis for GLUT4 expression in the mouse endometrium on pregnant days 1–4. Relative density analysis of GLUT4 by Image Lab 3.0 software (*n* = 3) normalized by β-actin. *Different superscript letters* (*a*, *b*, *c*) denote significant differences among groups (*P* < 0.05); the *same superscript letters* mean no significant difference (*P* > 0.05).

In order to elucidate the spatial and temporal distribution of GLUT4 in the mouse endometrium, the expression of GLUT4 in the mouse endometrium was first examined by immunohistochemistry (IHC) staining. GLUT4 was visualized in both the endometrial epithelium and stromal cells during days 1–4 of pregnancy. However, there were appreciable differences between the epithelial and stromal cells. On pregnant days 1–2, when estrogen dominated, GLUT4 was primarily expressed in the luminal epithelium and moderately in the subepithelial stroma. The distribution of GLUT4 spread to the stroma, and its expression scope and intensity were obviously expanded on pregnant days 3–4, when the uterus was gradually entering the receptive status under the actions of increasing progesterone and a small amount of estrogen (Figure 1A). Western blotting analysis revealed that the abundance of GLUT4 protein in the mouse uterus on pregnant days 1–4 was gradually increased, with a peak level on pregnant day 4 (Figure 1B), when the endometrial receptive status begins to be established, the “window of implantation.” GLUT4 protein expression and distribution were altered within the uterine tissues depending on days of mouse pregnancy, which suggests that GLUT4 expression varies temporally and spatially in response to the changes of ovarian hormones on pregnant days 1–4. This potentially indicates that it is important during this period.

### Ovarian Hormones Affect GLUT4 Expression in the Endometrial Epithelium

**FIGURE 2:**
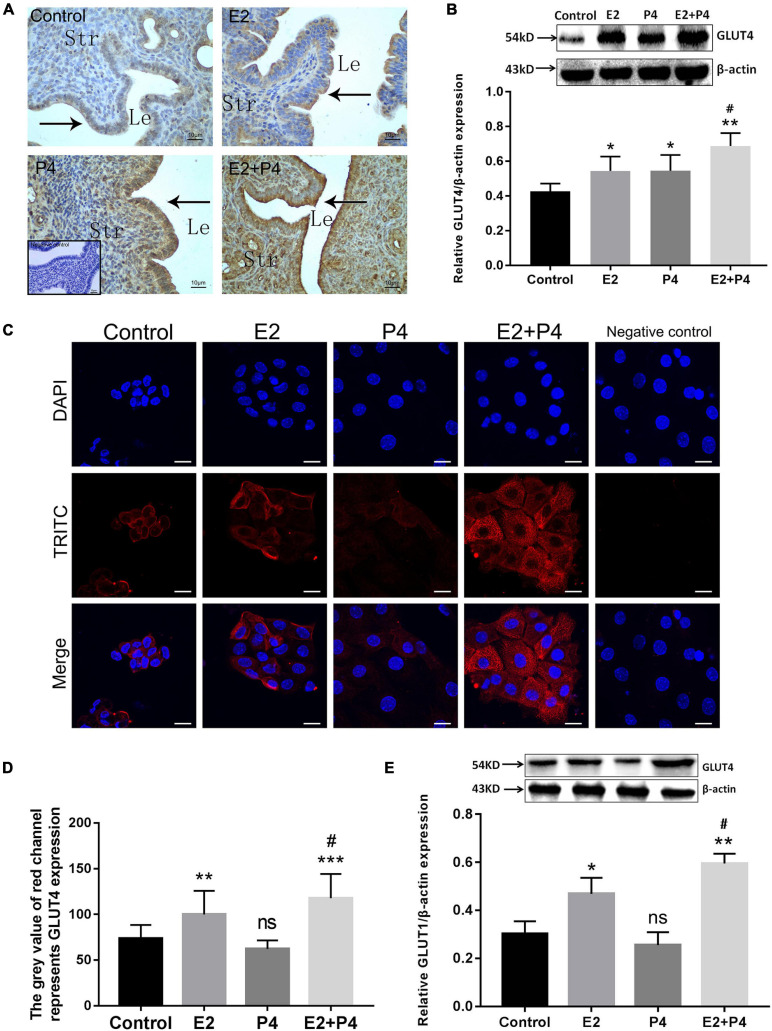
Estrogen and progesterone regulate the expression of GLUT4 in the endometrial epithelium. Mouse uteri were collected at 24 h after the last injection. One side of the uterine horns was fixed in 4% paraformaldehyde for paraffin blocks and the other side was stored at −80°C for Western blotting analysis, *n* = 10 per group. **(A)** Immunohistochemistry (IHC) staining for GLUT4 expression in ovariectomized (OVX) mouse endometrium 2 days after hormone treatments. Control (oil), E2 (0.2 μg/day estrogen), P4 (4 mg/day progesterone), and E2 + P4 (0.2 μg/day E2 + 4 mg/day P4). Negative control is without the primary antibody (shown at the *bottom of the left-hand corner*). *Le*, luminal epithelium; *Str*, stromal tissue. *n* = 10 per group. *Scale bar*, 10 μm. **(B)** Western blotting analysis for GLUT4 expression in OVX mouse uterine tissues (*n* = 3, normalized by β-actin). **(C)** Immunofluorescence staining for GLUT4 expression in mouse endometrial epithelial cells (EECs) 48 h after hormone treatments: control (solvent control), E2 (10^–8^ mol/L), P4 (10^− 6^ mol/L), and E2 + P4 (combination of 10^–8^ mol/L E2 and 10^− 6^ mol/L P4). Negative control is without the primary antibody. The experiment was performed at least three times. Images were taken at × 40. *Scale bar*, 10 μm. **(D)** Quantitative analysis of immunofluorescence staining using Image-Pro Plus 6.0 software. **(E)** Western blotting analysis for GLUT4 expression in mouse EECs. The cells were harvested 48 h after hormone treatments. Relative density analysis of the GLUT4 proteins by Image Lab 3.0 software (*n* = 3, normalized by β-actin). *ns*, not significant. *P < 0.05, **P < 0.01, ***P < 0.01 *vs.* control; ^#^*P* < 0.05 *vs.* the E2 group.

In order to examine the effects of ovarian hormones on GLUT4 expression in the mouse endometrium, *in vivo* experiments were conducted using ovariectomized (OVX) mice as the model. GLUT4 expression in the OVX mouse endometrium was detected by IHC and Western blotting. The results of IHC showed that GLUT4 expression was very low in the control group and that E2 increased the expression of GLUT4 in the endometrial epithelium of OVX mice, while the expression of GLUT4 in mouse uteri was further increased in the 0.2 μg/day E2 + 4 mg/day P4 (E2 + P4) group (Figure 2A). In addition to the expression levels, strong GLUT4 immunostaining was also observed on the apical surface of the luminal epithelium in the E2 + P4 group. Western blotting analysis also revealed that the level of GLUT4 protein was higher in the E2 + P4 group than that in the control or the E2 group (*P* < 0.05; Figure 2B). *In vitro*, primary mouse EECs were used as the model. The purity of mouse EECs was evaluated by immunofluorescence (IF) staining using CK-19 as the epithelial cell marker. The results showed that the EECs grew very well with high purity (positivity rate of CK-19 > 90%; Supplementary Figure 1), which was used for subsequent experiments *in vitro*. The expression of GLUT4 in mouse EECs was detected by IF and Western blotting. The results of IF showed that GLUT4 was mainly localized in the cell membrane and cytoplasm in mouse EECs. The expression of GLUT4 was obviously increased in the E2 group than in the control group, and it was higher in the E2 + P4 group than that in the E2 group, while P4 alone cannot induce GLUT4 expression in the EECs *in vitro* (Figures 2C,D). The results of Western blotting also revealed that E2 alone or E2 + P4 induced the expression of GLUT4 in the EECs, while the expression of GLUT4 in the P4 group was not significantly increased (*P* > 0.05; Figure 2E). These results suggest that both E2 and P4 can induce GLUT4 expression, but in terms of endometrial epithelial cells, E2 is a key factor that enhances GLUT4 expression in epithelial cells because, in the presence of E2, such as in the E2 group or the E2 + P4 group, the expression and the localization of GLUT4 in epithelial cells were obviously increased *in vivo* or *in vitro*.

### GLUT4 Affects Glucose Uptake in Mouse Endometrial Epithelial Cells

**FIGURE 3:**
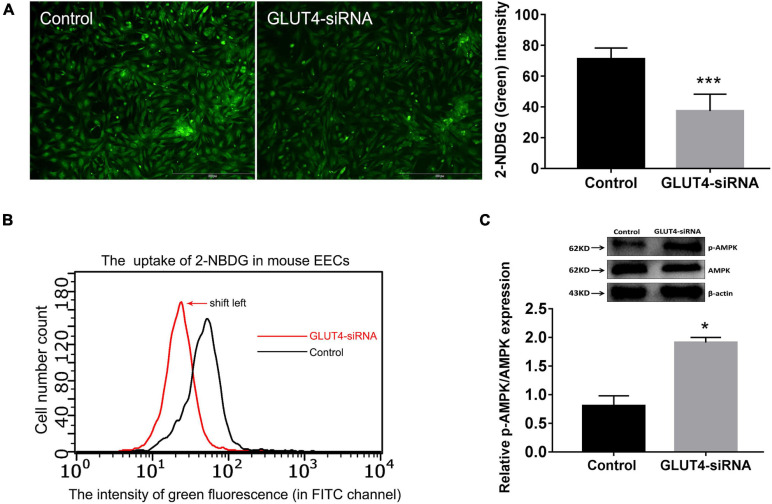
Effect of GLUT4 on glucose uptake in mouse endometrial epithelial cells (EECs) following GLUT4-siRNA transfection. **(A)** The 2-NBDG (*green*) signals in mouse EECs shown by fluorescent scope. The cells in five visual fields per group were counted and photographed. The intensity of 2-NBDG in mouse EECs was analyzed by Image-Pro Plus 6.0 software. ***P < 0.001. *Scale bar*, 500 μm. **(B)** The uptake of 2-NBDG in mouse EECs was detected by microcapillary flow cytometry (Millipore, United States) at the FITC channel. The *abscissa* is the fluorescence intensity and the *ordinate* is the cell number. The curve shift *to the left* indicated a dropped glucose uptake in mouse EECs. **(C)** The expressions of p-AMPK and AMPK in mouse EECs were detected by Western blotting. The ratio of p-AMPK/total AMPK reflects the state of intracellular energy demand in mouse EECs (the ratio increases as the intracellular energy demand increases). The relative densities of the p-AMPK and AMPK proteins were normalized by β-actin. *P < 0.05 *vs.* control. The experiment was performed at least three times.

Firstly, GLUT4-siRNA (100 nM) was transfected into mouse EECs. The *in vitro* interference efficiency of GLUT4-siRNA was evaluated by determining the expression of GLUT4. The results of Western blotting showed that the levels of GLUT4 protein in mouse EECs were obviously decreased after GLUT4-siRNA transfection (Supplementary Figure 2B). The green signals visibly confirmed that GLUT4^FAM^ -siRNA was successfully transfected into the EECs (Supplementary Figure 2C). Then, 2-NBDG was used as an indicator of glucose uptake and glucose uptake assay was performed 48 h after GLUT4-siRNA transfection. Fluorescence microscopy showed that the uptake of 2-NBDG in mouse EECs was visibly decreased in the GLUT4-siRNA group when compared to the control group (Figure 3A). Flow cytometry detection also revealed that the curve of 2-NBDG uptake shifted to the left, indicating that the glucose uptake in mouse EECs decreased in the GLUT4-siRNA group (Figure 3B). Next, we further evaluated the effect of GLUT4 on energy metabolism in mouse EECs. 5′-AMP-activated protein kinase (AMPK) is an important intracellular energy sensor. When the intracellular energy demand increases, more AMPK is activated by phosphorylation (Turner et al., 2016; Lin and Hardie, 2018). Therefore, the ratio of p-AMPK/total AMPK was used to reflect the state of intracellular energy demand (the ratio increases as the intracellular energy demand increases). The results of Western blotting revealed that the ratio of p-AMPK/total AMPK in the GLUT4-siRNA group was significantly increased compared to the control (Figure 3C), which indicates that the downregulation of GLUT4 affects the stability of energy metabolism in mouse EECs by insufficient glucose intake. All the above results indicated that GLUT4 is an important transporter that mediates glucose uptake in mouse EECs.

### GLUT4 in Mouse Endometrial Epithelium Helps Maintain Glucose Concentration in the Uterine Fluid and Endometrial Receptivity Related to Implantation

**FIGURE 4:**
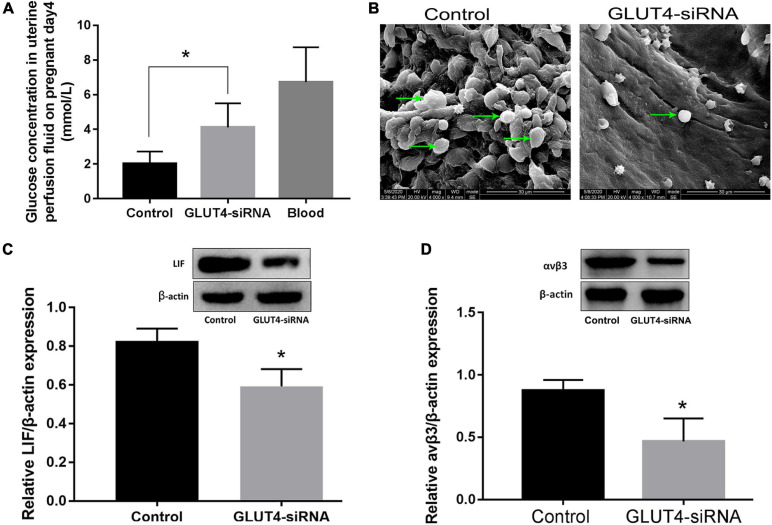
Effect of GLUT4 on glucose concentration in the uterine fluid and endometrial receptivity following GLUT4-siRNA transfection. **(A)** Uterine fluid was collected on pregnant day 4 using *in vivo* uterine perfusion and glucose concentration in the uterine fluid was measured by HPLC. The glucose concentration in the GLUT4-siRNA side was obviously higher than that in the control side (4.12 ± 0.57 *vs.* 2.01 ± 0.29 mM, ^∗^*P* < 0.05 *vs.* control, *n* = 12), but was still lower than that in blood (6.7 ± 0.72 mM). **(B)** Pinopode formation in the luminal epithelium was observed by SEM on pregnant day 4. *Arrows* indicate pinopodes. *Scale bar*, 30 μm. **(C)** Western blotting analysis for leukemia inhibitory factor (LIF) expression on pregnant day 4 following GLUT4-siRNA transfection. Relative density analysis of the LIF protein by Image Lab 3.0 software (*n* = 3, normalized by β-actin). **(D)** Western blotting for integrin ανβ3 expression following GLUT4-siRNA transfection (*n* = 3, normalized by β-actin). *P < 0.05 *vs.* control.

In order to study the effect of GLUT4 in the endometrial epithelium on maintaining the glucose concentration in the uterine fluid in mice, on pregnant day 2, one side of the uterine horns was treated with 25 μl GLUT4-siRNA (500 μM) and the other side was treated with the same amount of non-specific siRNA as the control. The *in vivo* interference efficiency of GLUT4-siRNA was evaluated by determining the expression of GLUT4. GLUT4 immunostaining was obviously decreased in the GLUT4-siRNA side than in the control side (Supplementary Figure 3A), and this result was also validated on protein expression by Western blotting (Supplementary Figure 3B). The green signals visibly confirmed that GLUT4^FAM^-siRNA was successfully transfected into the endometrial epithelium *in vivo* (Supplementary Figure 3C). Next, the uterine fluids on pregnant day 4 were collected by *in vivo* uterine perfusion and the glucose concentration in uterine fluids was measured by high-performance liquid chromatography (HPLC) (Supplementary Figure 4). The results of HPLC showed that the glucose concentration in the GLUT4-siRNA side (4.12 ± 0.57 mM) was significantly higher than that in the control side (2.01 ± 0.29 mM), but it was still lower than that in the blood (6.7 ± 0.72 mM) (Figure 4A). This observation indicated that GLUT4 in the endometrial epithelium affects the glucose concentration in uterine fluids. Meanwhile, the uterine tissues were also collected on pregnant day 4 to analyze the expressions of the endometrial receptivity markers. Pinopode formation in the luminal epithelium on pregnant day 4 was observed with scanning electron microscopy (SEM). The results of SEM showed that the numbers of pinopodes were visibly reduced in the GLUT4-siRNA side compared to that in the control side (Figure 4B). The results of Western blotting showed that the expressions of LIF (Figure 4C) and integrin αvβ3 (Figure 4D) in the GLUT4-siRNA side were also significantly decreased. These results suggested that the downregulation of GLUT4 in the endometrial epithelium impaired uterine receptivity by inhibiting the expressions of the receptivity markers: pinopodes, LIF, and integrin ανβ3.

### GLUT4 in Mouse Endometrial Epithelium Affected Embryonic Development and Implantation

**FIGURE 5:**
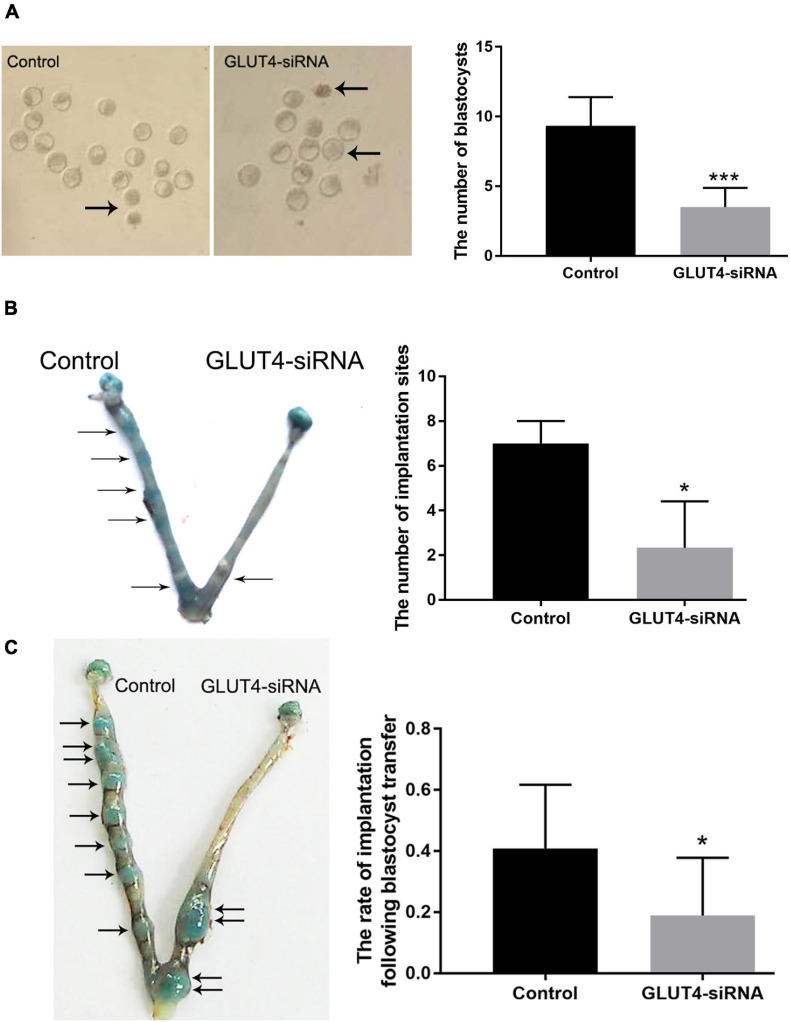
Effect of GLUT4 on embryonic development and implantation. **(A)** On pregnant day 4, embryonic development was observed. *Arrows* indicate abnormal blastocysts. The number of normal blastocysts in the GLUT4-siRNA side was obviously decreased compared to that in the control side (3.5 ± 0.6 *vs.* 9.3 ± 0.8, ***P < 0.001 *vs.* control, *n* = 12). **(B)** On pregnant day 5, the implantation site was observed. The number of implantation sites in the GLUT4-siRNA side was significantly decreased compared to that in the control side (2.3 ± 1.2 *vs.* 7 ± 0.6, *P < 0.05 *vs.* control, *n* = 12). **(C)** Embryonic implantation rates on pregnant day 5 of embryo transfer. Eight blastocysts from donor mice were transferred into one uterine horn of each recipient mouse. The implantation rate in the GLUT4-siRNA side was significantly lower than that in the control side (0.19 ± 0.07% *vs.* 0.41 ± 0.07%, *P < 0.05 *vs.* control, *n* = 12).

We further explored the effects of GLUT4 on embryonic development and implantation. The results showed that the normal blastocyst number on pregnant day 4 was significantly decreased in the GLUT4-siRNA side (3.5 ± 0.6) compared to that in the control side (9.3 ± 0.8) (Figure 5A), and the implantation number on pregnant day 5 was also obviously decreased in the GLUT4-siRNA side (2.3 ± 1.2) compared to the control side (7 ± 0.6) (Figure 5B). These results suggest that GLUT4 in mouse endometrial epithelium affected embryonic development and implantation. To eliminate the influence of embryos themselves on implantation, we carried out embryo transfer experiments. The results showed that the rate of implantation in the GLUT4-siRNA side (0.19 ± 0.07%) was significantly lower than that in the control side (0.41 ± 0.07%; Figure 5C), which further indicated that GLUT4 in mouse endometrial epithelium was involved in implantation.

## Discussion

The uterine epithelium includes the luminal epithelium (LE) and glandular epithelium (GE), which extends from the LE into the stromal layer. The LE is the first maternal contact for an implanting embryo and serves as the transient gateway for embryo development and subsequent implantation in the uterus. In this process, LE cells have to go through sequential changes during early pregnancy, e.g., proliferation and differentiation (Aplin and Ruane, 2017), cytoskeleton remodeling (Tung et al., 1988; Kalam et al., 2018), vesicle trafficking (Li et al., 2020; Rai et al., 2021), secretion and resorption of uterine fluid contents (Azkargorta et al., 2018; Exposito et al., 2018; Matorras et al., 2020), etc., to reach into a receptive state that supports embryo implantation. All these LE changes are energy-consuming processes, which undoubtedly depend on glucose uptake and metabolism. Glucose, one of the energy substances in uterine fluid, is essential not only for intrauterine embryo growth but also for preparing a receptive endometrium for implantation. Before full invasion of trophoblast and the establishment of a functional placenta, glucose, as a major nutrient for embryos and cells of the uterus, must be adequately transported from maternal circulation by the facilitative glucose transporters, commonly known as GLUTs (Augustin, 2010; Mueckler and Thorens, 2013), because neither embryo nor endometrium carries out gluconeogenesis (Zimmer and Magnuson, 1990; Yánez et al., 2003).

GLUT4 is one of the glucose transporters that are involved in rapid glucose uptake among various kinds of cells to contribute to glucose homeostasis (Huang and Czech, 2007; Klip et al., 2019). We suspect that GLUT4 is involved in maintaining the glucose concentration in the uterine fluid or establishing uterine receptivity, which are essential for subsequent embryonic development and implantation. Firstly, the obtained results demonstrates that GLUT4 expression in the mouse endometrium is in a spatiotemporal manner due to the changes of ovarian hormones on days 1–4 of pregnancy and that GLUT4 is mainly localized in the LE and GE cells, which are in line with previous observations (Gao et al., 2009; França et al., 2015). As shown in Figure 1A, on pregnant day 4, when a small amount of E2, along with increased P4, induces a transient receptive state of the endometrium, the “window of implantation” (Cha et al., 2012; Kim and Kim, 2017), the abundance of GLUT4 protein in mouse uteri reached a peak. There is no doubt that GLUT4 is one regulatory target of ovarian estrogen and progesterone, and we deduce that the spatiotemporal expression of GLUT4 regulated by E2 and P4 may be indicative of its protein’s function during the window of implantation. Estrogen and progesterone have long been known to affect glucose metabolism and utilization through the regulation of some GLUT expressions, although reports regarding ovarian hormones directly regulating GLUT4 expression in the uterus are limited, especially under physiological conditions (Kim and Moley, 2009; França et al., 2015). For example, a recent study showed that progesterone induces GLUT1 expression and that knockdown of GLUT1 may cause endometrial dysfunction by affecting glucose metabolism, thereby interfering with embryo implantation (Zhang et al., 2020). In addition, estrogen can directly induce GLUT4 expression (Barros et al., 2008; Barreto-Andrade et al., 2018; Fatima et al., 2019) or indirectly enhance the membrane translocation of insulin stimulation to increase glucose uptake in some insulin-sensitive tissues, such as skeletal muscle cells and adipocytes (Furtado et al., 2002; Leto and Saltiel, 2012; Alam et al., 2016). But the uterine tissue is not a typical insulin-sensitive tissue, despite insulin receptor and insulin-like growth factors being expressed in the uterus (Zhou et al., 1994; Mioni et al., 2012). Whether such a mechanism exists in the uterus is not clear. In our study, E2 appears to be a key factor that enhances GLUT4 expression in epithelial cells because, in the presence of E2 such as in the E2 group or the E2 + P4 group, the expression and the localization of GLUT4 in endometrial epithelial cells are obviously increased *in vivo* or *in vitro*. This is also consistent with previous studies (Ma et al., 2003) suggesting that a small amount of estrogen during the window of implantation is a critical determinant in specifying the duration of uterine receptivity. Thus, we speculate that a higher GLUT4 expression on pregnant day 4, when the physiological effects of increasing progesterone and a small amount of estrogen on uterine tissues have reached a peak, may be one way of adapting to more glucose demand in interactions between the endometrial epithelium and the embryo during the window of implantation.

In our study, GLUT4, which participates in epithelium glucose uptake and transport, indeed is pivotal to embryo development and implantation during the window of implantation. Firstly, GLUT4 has been shown to be an important transporter that mediates glucose uptake in mouse EECs *in vitro* (Figure 3). Glucose concentration of the uterine fluid in the GLUT4-siRNA side on pregnant day 4 was increased compared to that in the control side, but it was still lower than that in the blood, suggesting that GLUT4 plays a vital role in maintaining glucose concentration in the uterine fluid. It also indicates that the uterine fluid is a low-glucose environment, which is consistent with the research of Harris et al., which also reported that glucose in the uterine fluid is 7–25 times lower than that in the blood (Harris et al., 2005). In fact, in humans and ruminants, the components of the uterine fluid, including glucose, are eventually derived from the blood (Salleh et al., 2005; Satterfield et al., 2010; Bazer et al., 2012). But many nutrients in the uterine fluid including glucose are very different from those in the blood, speculating that these differences are most likely due to barriers of the LE cell layer and to specific transporters in the LE (Gao et al., 2009; Chan et al., 2012; Greening et al., 2016). Considering that P4 can decrease the tight-junction barrier function of the endometrial epithelium during pregnancy, this likely permits an increase in the paracellular trafficking of blood-derived glucose into the uterine lumen (Orchard and Murphy, 2002; Satterfield et al., 2007; Jalali et al., 2020) and reduced the volume of water in the uterine fluid when the uterine cavity was gradually closed (Lu et al., 2013); thus, it is difficult to conclude that an increased GLUT4 expression accounts for the greater amounts of glucose in the uterine fluid on pregnant day 4. But, in our study, glucose concentration in the uterine fluid depends, at least partly, on GLUT4’s function because it was obviously increased due to a dysfunction of GLUT4 in the endometrial epithelium.

In mice, embryos enter the uterine cavity on the morning of pregnant day 3, and the endometrium becomes receptive by the afternoon of day 4. Once embryos enter the uterine cavity, they begin to use glucose as their preferred energy source (Riley and Moley, 2006). Our observations suggest that the spatiotemporal expression of GLUT4 in the mouse endometrium is adaptive for embryos utilizing glucose as the preferred energy source. Some studies have shown that embryos demand different energy substances at different phases. Glucose inhibits embryo development at the early stage (cleavage stage) while promoting it at a later stage; therefore, oocytes or early embryos use pyruvate rather than glucose as their energy substance (Conaghan et al., 1993; Hugentobler et al., 2008; Mullen et al., 2014). Obviously, in order to adapt to the embryo’s demand for glucose, the glucose concentration in the uterine fluid possibly fluctuated with the spatiotemporal expression of GLUT4 in the mouse endometrium (Harris et al., 2005). In our study, although the glucose in the uterine fluid was still lower than that in the blood, the downregulation of GLUT4 by GLUT4-siRNA resulted in an increased glucose concentration in the uterine fluid on pregnant day 4, and this abnormal alteration rendered the uterine environment detrimental to pre-implantation embryonic development and then impaired implantation. Evidences for the detrimental effect of a high glucose concentration on embryo development have been documented in several species including humans (Scott-Drechsel et al., 2013), mice (Fraser et al., 2007), rats (Baack et al., 2014), and bovine (Jiménez et al., 2003). Thus, a strict intrauterine environment, including glucose that must be kept within an optimal range, is necessary for embryo development. In this study, specific GLUT4 expression, especially in LE cells, is vital to safeguard pre-implantation embryo growth in the uterine cavity by maintaining proper glucose concentration in the uterine fluid. To be true, we cannot completely rule out other cell types, especially the GE’s contribution to the intrauterine fluid, but its contribution to intrauterine fluid resorption may be limited due to its minimal contact with the intrauterine fluid, and GE-deficient mice show no obvious intrauterine fluid accumulation during peri-implantation (Kelleher et al., 2016). Thus, the LE possibly contributes mainly to the uterine fluid microenvironment, including glucose, through balance of the endometrial epithelium’s active secretion and reabsorption (Xie et al., 2018; Ye, 2020).

Besides, adequate glucose uptake and metabolism in the endometrial epithelium, especially in the LE (Evans et al., 2020), are essential for establishing endometrial receptivity because embryo entering the uterine cavity must first attach to the LE and then initiate implantation. Thus, functional defects of the LE may directly affect embryo recognition, adhesion, and subsequent implantation. Under the influences of ovarian hormones, estrogen and progesterone, the LE undergoes great changes during early pregnancy, such as proliferation and differentiation, cytoskeleton remodeling, secretion and resorption of uterine fluid contents, etc., to reach into a receptive state, and all these LE changes are energy-consuming processes and inevitably dependent on the glucose supply (Ye, 2020). Therefore, defective glucose uptake of the LE due to the dysfunction of GLUTs may directly affect its cellular metabolic and functional activities during the window of implantation (Zhang et al., 2020). Pinopodes are considered as morphological indicators of the establishment of uterine receptivity (Murphy, 2000; Kazachkov et al., 2019). Upon embryo attachment, the LE surface becomes smoother, and this process is closely related to cytoskeleton remodeling and the movement of the LE, which are inevitably dependent on the supply of energy. The LIF is strongly expressed in the receptive uterus, and its deficiency will lead to implantation failure (Stewart et al., 1992). Integrin ανβ3, one of the adhesion-promoting molecules, was demonstrated to be expressed in endometrial epithelial cells during the receptive phase (Lessey and Arnold, 1998). Studies have shown that the expressions of adhesion molecules in endometrial epithelial cells increase at the receptive stage (Fujiwara et al., 2020), and most of which are transmembrane glycosylated modified proteins, while the glycosylated precursors, such as UDP-GlcNAc and UDP-GalNAc, are mostly derived from the branches of glucose metabolism. This also suggests a role of glucose metabolism in regulating epithelial cell functions. Recently, integrins ανβ3 and ανβ5 were reported to be necessary for the LIF-mediated adhesion of trophoblast cells to the endometrial epithelium during embryonic implantation (Chung et al., 2016). Thus, LIF and integrin ανβ3 are used as indicators of the establishment of uterine receptivity. In our study, the downregulation of GLUT4 resulted in the defective glucose uptake of endometrial epithelial cells, which further led to impaired endometrial receptivity by inhibiting pinopode formation and the expressions of endometrial receptivity markers, LIF and integrin ανβ3, and then impaired implantation (Franasiak et al., 2014; Chung et al., 2019). In embryo transfer experiments, pretreatment of one side of the mouse uterine horns with GLUT4-siRNA also resulted in a reduced implantation rate, even if excellent embryos were selected for transfer. Therefore, dysfunction of the endometrial epithelium caused by reducing the expression of GLUT4-mediated insufficient glucose uptake is also an important cause of implantation failure.

Our evidence confirms that a higher GLUT4 expression during the window of implantation is required for embryonic development and implantation, which is helpful in maintaining proper glucose concentration in the uterine fluid for embryo development and establishing endometrial receptivity for implantation. Our investigations are the first to link the critical role of GLUT4 in the endometrial epithelium glucose uptake and transport to the uterine fluid glucose concentration and endometrial receptivity during early pregnancy, which may provide new clues for understanding the pathogenesis of infertility, such as PCOS, endometrial cancer, and early miscarriage.

## Materials and Methods

### Ethics Statement

This study was approved by the Animal Use and Care Committee of Sichuan University. All experimental and surgical procedures were in compliance with the recommendations of the National Institute of Health Guide for the Care and Use of Laboratory Animals (NIH publication no. 80-23), revised in 2010. All efforts were exerted to minimize suffering of the mice.

### Pregnancy Mouse Model

Female CD-1 mice (8–10 weeks old, *n* = 10 per group) were mated with fertile males of the same strain to achieve pregnancy (day 1 = day of observation of the vaginal plug). Embryos could be recovered from the oviducts and uteri on pregnant days 1–4 to further confirm the pregnancy. Animals were sacrificed by cervical dislocation and the uterine tissues were collected at 2,000 h on the different days of pregnancy. Some of the tissues were stored at −80°C for Western blotting analysis; the remaining uterine tissues were fixed in 4% paraformaldehyde for immunohistochemistry (IHC) staining. Implantation sites were demarcated by discrete blue bands through tail intravenous injection of 0.1 ml 1% Chicago blue on pregnant day 5 or day 6.

### Ovariectomized Mouse Model

Female CD-1 mice (6 weeks old) were ovariectomized *via* dorsal incision under 1% (*w*/*v*) pentobarbital sodium (100 mg/kg) anesthesia. To determine the effects of ovarian hormones on GLUT4 expression in the mouse endometrium, different hormone treatments were administered *via* subcutaneous injection behind the neck cuff 2 weeks after ovariectomy. Progesterone (P0130, Sigma) and estradiol-17β (E8875, Sigma) were dissolved in sesame oil. Ovariectomized mice (*n* = 10 per group) were randomly divided into four groups, which received the following hormone treatments: (i) control group: 2 days treatment with sesame oil, which acts as the control; (ii) E2-treated group: 0.2 μg estradiol-17β (E2) in a final volume of 100 μl each day for two consecutive days; (iii) P4-treated group: 4 mg progesterone (P4) for 2 days; (iv) E2 + P4-treated group: a combination of E2 (0.2 μg) + P4 (4 mg) for 2 days. Uteri from these mice were collected 24 h after the last injection. Some of the tissues were stored at −80°C for Western blotting analysis; the remaining uterine tissues were fixed in 4% paraformaldehyde for IHC.

### Endometrial Epithelial Cell Model

Endometrial epithelial cells (EECs) were enzymatically isolated from the mouse uterine tissues on pregnant day 4 in accordance with the previously described method (Pan et al., 2017). The EECs were cultured with complete media consisting of phenol red-free Dulbecco’s modified Eagle’s medium (DMEM)/F12 supplemented with charcoal-stripped 10% fetal bovine serum (FBS). The purity of the EECs was confirmed by checking cytokeratin-19 as an epithelial cell marker *via* immunofluorescence staining.

To prove the effects of ovarian hormones on the expression of GLUT4 in the EECs, the EECs were cultured in six-well plates according to the aforementioned methods. Progesterone and estradiol-17β were dissolved in absolute alcohol to obtain E2 (10^–6^ mol/L) and P4 (10^–4^ mol/L) stock solutions. The cells were divided into four groups, which received the following hormone treatments: (i) control group: the cells were treated with the same amount of solvent as the control; (ii) E2 group: the cells were treated with 10^–8^ mol/L E2; (iii) P4 group: the cells were treated with 10^–6^ mol/L P4; and (iv) E2 + P4 group: the cells were treated with 10^–8^ mol/L E2 combined with 10^–6^ mol/L P4. The cells were harvested 48 h after hormone treatments for immunofluorescence staining or Western blotting to detect GLUT4 expression. The experiments were performed on six-well plates with ≥ 80% cell confluence.

### Immunohistochemistry

Uterine tissue sections (5 μm) were rehydrated in a descending concentration of ethanol following antigen retrieval in citrate buffer (pH 6.0). The sections were treated according to the manufacturer’s standard protocol using SP-9001 detection kit (SP-9001, ZSGBBIO, China). After the endogenous peroxidase activity was quenched with 0.3% H_2_O_2_, the sections were blocked in FBS at 37°C for 30 min. Then, the sections were incubated in rabbit anti-GLUT4 antibodies (1:500 dilution; ab33780, Abcam, United States) overnight at 4°C. Immunodetection was carried out with the diaminobenzidine–horseradish peroxidase (HRP) reaction system and counterstained with hematoxylin for nuclear staining. The negative control received the same procedure, except that the primary antibody was replaced with phosphate-buffered saline (PBS). Images were analyzed and processed using Image-Pro Plus 6.0 software.

### Western Blotting Analysis

Total protein extracts from the uterine tissues or the EECs were lysed with ice-cold RIPA protein lysis buffer (P0013B, Beyotime). The protein concentrations were detected by the BCA protein assay (P0012S, Beyotime). Protein was loaded into the lanes of SDS-PAGE gels for Western blotting, which was performed according to a standard protocol. An equal amount of protein (40 μg protein per sample) from the samples was mixed with a loading dye and separated using 12% SDS-PAGE, then transferred into a nitrocellulose filter membrane (Millipore, United States). The membranes were blocked with 5% skim milk at room temperature for 2 h and incubated with rabbit anti-GLUT4 antibodies (1:1,000 dilution; ab33780, Abcam, United States), AMPK (1:600; ab131512, Abcam), *p*-AMPK (1:600; ab23875, Abcam), rabbit anti-integrin ανβ3 antibody (1:1,000 dilution; bs-1310R, Bioss, China), rabbit anti-LIF antibody (1:1,000 dilution; ab101228, Abcam, United States), and rabbit anti-β-actin (1:2,000 dilution; AF7018, Affinity, China) overnight at 4°C. The signal was detected with HRP-conjugated secondary antibody (1:2,000; bs-0295G-HRP, Bioss, China) for 1 h at room temperature and visualized by enhanced chemiluminescence reagents (SW2030, Solarbio, China). Photos of the blots were captured by a gel documentation system, and the density of each band was determined using Image Lab 3.0 software. The ratio of each target band/β-actin was calculated and was considered as the expression level of the target proteins.

### Immunofluorescence Staining

Briefly, the EECs were seeded on polylysine-coated glass coverslips to increase the attachment of cells to the glass coverslips. The cells were washed with PBS and fixed with 4% paraformaldehyde for 10 min after different treatments. This was followed by permeabilization with 0.2% Triton X-100. After blocking for 30 min in 3% (*w*/*v*) bovine serum albumin (BSA) at 37°C, the cells were incubated with anti-GLUT4 (1:400) at 4°C following exposure to TRITC-conjugated secondary antibodies (1:800 dilution in PBS). The cells were counterstained with DAPI for nuclear staining and the fluorescence was observed under a Zeiss confocal microscope. Images were analyzed and processed using Image-Pro Plus 6.0 software.

### Downregulation of GLUT4 by siRNA *in vitro* or *in vivo*

*In vitro*, the mouse EECs were directly seeded in six-well or 12-well plates supplemented with 17β-estrogen (10^–8^ mol/L) to promote ≥ 80% cell confluence within 24 h. Briefly, according to the protocol of RNAi-Mate Transfection Reagent, siRNA or the RNAi-Mate Reagent was dissolved in serum-free DMEM/F12 and incubated at room temperature for 5 min. The two were mixed and incubated at room temperature for 30 min to form a siRNA/RNAi-Mate mixture. The mixture was added to the EECs at a final concentration of GLUT4-siRNA of 100 nM. Glucose uptake assay was performed 48 h after GLUT4-siRNA transfection.

*In vivo*, on pregnant day 2, prior to blastocysts entering the uterine cavity, 25 μl of the siRNA/RNAi-Mate mixture (the final concentration of GLUT4-siRNA was 500 μM) was directly injected into one side of the mouse uterine horns and, with the other side injected with the same amount of non-specific siRNA as the control, followed by comparison of the changes of glucose concentration in the uterine fluid, the expressions of key uterine receptivity markers, embryonic development, and implantation on pregnant day 4 or day 5.

The *in vitro* or *in vivo* interference efficiency of GLUT4-siRNA was evaluated by determining the expression of GLUT4. Small interfering RNAs specific for GLUT4 with cholesterol modification, referred to as GLUT4-siRNA, were designed and synthesized by GenePharma Company (Shanghai, China). The non-specific siRNA control can be used in conventional parallel control experiments. Fluorescent-labeled GLUT4^FAM^-siRNA can be observed under a fluorescence microscope, which is helpful for optimizing the transfection conditions and monitoring the transfection efficiency *in vitro* or *in vivo*. GLUT4-siRNA, non-specific siRNA, GLUT4^FAM^-siRNA, and the RNAi-Mate Transfection Reagent were all purchased from GenePharma Company.

### Glucose Uptake Assay

Glucose uptake by the EECs was measured using a fluorescently labeled deoxyglucose analog, 2-NBDG, as a fluorescent probe, and the operation is as follows. The experiments were performed on six-well plates with ≥ 80% cell confluence. The cells were treated with GLUT4-siRNA for 48 h according to the aforementioned methods. The same amount of non-specific siRNA was used for the control. The transfection media were removed from each well and replaced with a 2-NBDG incubation buffer (200 μM 2-NBDG in HBSS buffer) and incubated for 30 min at 37°C, and then the 2-NBDG incubation buffer was removed from each well. The cells were washed with cold PBS and were directly observed using a fluorescence microscope, or were digested and collected. Of the cell suspension (in HBSS buffer), 300 μl was analyzed by microcapillary flow cytometry (Millipore, United States) at the FITC channel. With fluorescence intensity as the abscissa and cell number as the ordinate, 1 × 10^4^ cells per group were counted to plot the glucose uptake curve of 2-NBDG.

### Uterine Fluid Collected by *in vivo* Uterine Perfusion

*In vivo* uterine perfusion was performed in accordance with two previously described methods, with some modifications (Gholami et al., 2013; Xie et al., 2018). Briefly, on pregnant day 2, 25 μl GLUT4-siRNA was directly injected into one side of the mouse uterine horns and the other side injected with the same amount of non-specific siRNA as the control. On pregnant day 4, the female mouse was anesthetized with intraperitoneal injection of 1% (*w*/*v*) pentobarbital sodium (100 mg/kg) and placed on a heat pad to maintain the body temperature at 37°C. An incision was made to expose the abdominal cavity. An in-going tube pre-filled with medical saline was inserted at the distal end of the uterine horn and an out-going tube was inserted at the utero-cervical junction to collect the uterine fluid. A syringe-driven infusion pump with double channels (Harvard Apparatus 4501, United States) was used to deliver medical saline into the duplex uterine lumens at a constant rate of 1 μl/min for 2 h to collect the uterine perfusion fluid into a small polythene tube covered with parafilm to minimize evaporation. At the end of uterine perfusion, anesthetized animals were sacrificed by cervical dislocation. Glucose concentration in the uterine perfusion fluid was detected by HPLC and compared. The uterine tissues were also collected and fixed in 4% paraformaldehyde for IHC or for Western blotting analysis.

### High-Performance Liquid Chromatography

Firstly, 0.1, 0.25, 0.5, 1, 2, 4, 6, 8, and 10 mM of the glucose standard solution were prepared, and then the absorbance curves of the different glucose standard solutions were detected by HPLC. A standard curve was established by different glucose standard solutions with their corresponding absorbance values, and this standard curve was used to calculate the glucose concentration in the samples. The samples were processed as follows. Briefly, 70 μl of the samples was mixed with 2 μl KH_2_PO_4_ (1 mol/L), 3 μl benzoyl chloride, and 15 μl NaOH (8 mol/L), following vortex oscillation for 5 min, then 15 μl H_3_PO_4_ (1.4 mol/L) and 100 μl ethyl acetate were added, following vortex oscillation for 5 min. Centrifugation was done at 13,000 rpm for 5 min, and then 50 μl supernatant was taken and air-dried at room temperature. The white powder on the bottom of the polythene tube is benzoylated glucose. Next, a ratio of 7:3 acetonitrile to water was added to dissolve the powder, and then the glucose concentrations in the samples were determined by HPLC with reference to the glucose standard solutions.

### Embryo Transfer

Pseudopregnant mice (*n* = 12) were produced by mating females with vasectomized males. Female mice presenting with a vaginal plug were considered as pseudopregnant day 1. On pseudopregnant day 2, 25 μl GLUT4-siRNA was directly injected into one side of the mouse uterine horns and the other side was injected with the same amount of non-specific siRNA as the control. Then, at 2,000 h on pregnant day 4, eight blastocysts from donor mice were transferred into one uterine horn of each recipient mice; the implantation rates on pregnant day 6 between the duplex uterus of the same recipient mice were compared. A trait of the duplex uterus in mouse is that it does not permit the *trans*-uterine migration of embryos from one horn to the other; hence, it is favorable for the study of endometrial receptivity: one pretreated uterine horn or the other horn is used as the control, followed by a comparison of the implantation rates.

### Detection of Pinopodes by Scanning Electron Microscopy

Uterine tissues from pregnant day 4 were fixed in 2.5% (*w*/*v*) glutaraldehyde overnight. The samples were washed twice with PBS and once with a 4% sucrose solution. After gradient alcohol dehydration and drying, the samples were observed under a scanning electron microscope. The experiment was performed three times.

### Statistical Analysis

All data are presented as the mean ± SD. GraphPad Prism 7.0 software was used to assess the differences between the experimental groups. Univariate ANOVA was performed according to a paired experiment design, and *P* < 0.05 was considered statistically significant.

## Data Availability Statement

The original contributions presented in the study are included in the article/Supplementary Material, further inquiries can be directed to the corresponding author/s.

## Ethics Statement

The animal study was reviewed and approved by Animal Use and Care Committee of Sichuan University.

## Author Contributions

YuL designed the research, mainly performed the experiments and analyzed the results, and wrote the manuscript. YiL and Y-CW performed the main experiments. X-HD and D-ZY performed detection with HPLC. L-CL analyzed the results of HPLC. L-XZ, Y-CW, D-ZY, and J-HZ discussed the results. X-QZ, Y-DM, YiL, and Z-HC revised the manuscript. LN and L-MY designed the research and approved the final version of the manuscript. All authors contributed to the article and approved the submitted version.

## Conflict of Interest

The authors declare that the research was conducted in the absence of any commercial or financial relationships that could be construed as a potential conflict of interest.

## Supplementary Material

Supplementary Figure 1 Purity identification of the mouse EECs. **(A)** Representative picture of the cultured mouse EECs. The results showed that the EECs grew very well. Scale bar: 100 μm. **(B)** The purity of EECs was evaluated by immunofluorescence using Cytokeratin-19 as an epithelial cells marker. Vimentin as a stromal cells marker is the negative control. The results showed that the EECs with high purity (positive for CK-19 > 90%) and negative for Vimentin. Scale bar: 100 μm.

Supplementary Figure 2 *In vitro* interference efficiency of GLUT4-siRNA in mouse EECs. Three pairs of GLUT4 small interfering RNAs (siRNAs), were designed and transfected separately into Ishikawa cells, and the interference efficiency of each pair were determined by qPCR (data not shown) and western blotting at 72 h post-transfection. The siRNA pair with the strongest inhibitory effect on GLUT4 expression was modified with cholesterol, and then used in subsequent *in vitro* and *in vivo* experiments. **(A)** GLUT4 protein expression in Ishikawa cells was inhibited over 80% with Seq.3 siRNA compared with controls at 72 h. GAPDH served as loading control. **(B)** Western blotting for GLUT4 protein expression in mouse EECs following GLUT4-siRNA transfection. **P* < 0.05 *vs.* Control. **(C)** The green FAM signals (>90%) in mouse EECs following GLUT4^FAM^-siRNA transfection. Scale bar, 500 μm. The experiment was repeated three times.

Supplementary Figure 3 *In vivo* efficiency interference of GLUT4-siRNA on pregnant day 4. **(A)** IHC for GLUT4 expression on pregnant day 4 following GLUT4-siRNA transfection. GLUT4 positive staining was analyzed by image Pro-Plus 6.0 software. Integrated optical density per area (IOD/Area). Negative control is without the primary antibody. Scale bars, 20 μm. **(B)** Western blotting for GLUT4 expression on pregnant day 4 following GLUT4-siRNA transfection. **(C)** The green signals confirmed that GLUT4^FAM^-siRNA was visibly transfect into the luminal epithelium on pregnant day 4. The white arrow indicates the luminal epithelium. Scale bars: 100 μm. **P* < 0.05, ***P* < 0.01 *vs.* Control. The experiment was repeated three times.

Supplementary Figure 4 Glucose concentration in uterine fluid detected by high performance liquid chromatography (HPLC). **(A)** Absorbance curve of different glucose standard solutions detected by HPLC. Characteristic peak of glucose in HPLC appeared at about 20 min. **(B)** Standard curve was established by glucose standard solutions with their HPLC absorbance values, and this equation was used to calculate glucose concentration in samples.
