# Supplementary figures and images for: GLUT4 in Mouse Endometrial Epithelium: Roles in Embryonic Development and Implantation
Source: Front Physiol. 2021 Jun 25;12:674924. doi: 10.3389/fphys.2021.674924 (PMC8267529; doi:10.3389/fphys.2021.674924)

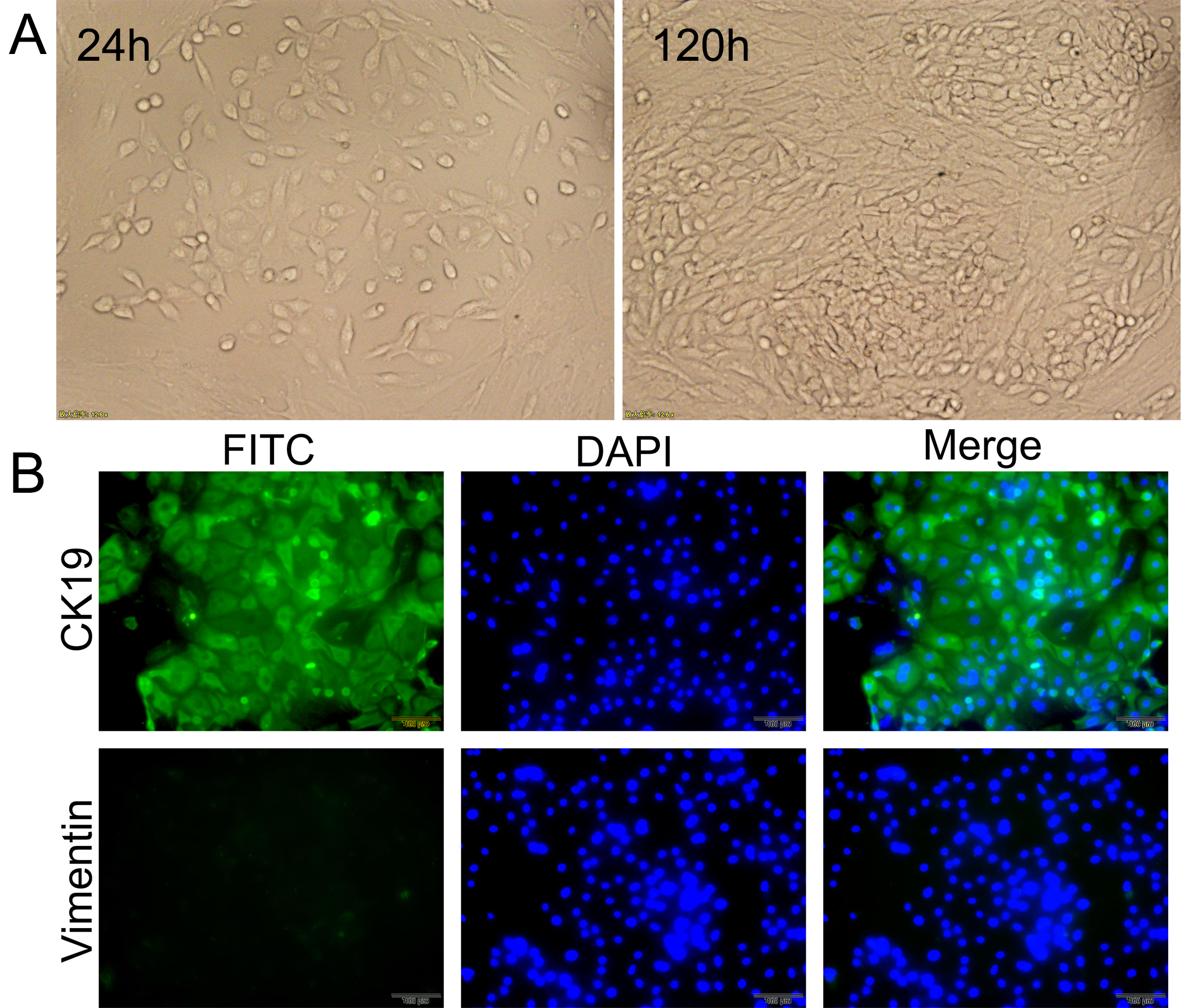

Supplement: Supplementary Figure 1 — Purity identification of the mouse EECs. (A) Representative picture of the cultured mouse EECs. The results showed that the EECs grew very well. Scale bar: 100 μm. (B) The purity of EECs was evaluated by immunofluorescence using Cytokeratin-19 as an epithelial cells marker. Vimentin as a stromal cells marker is the negative control. The results showed that the EECs with high purity (positive for CK-19 > 90%) and negative for Vimentin. Scale bar: 100 μm. [file Image_1.TIF]

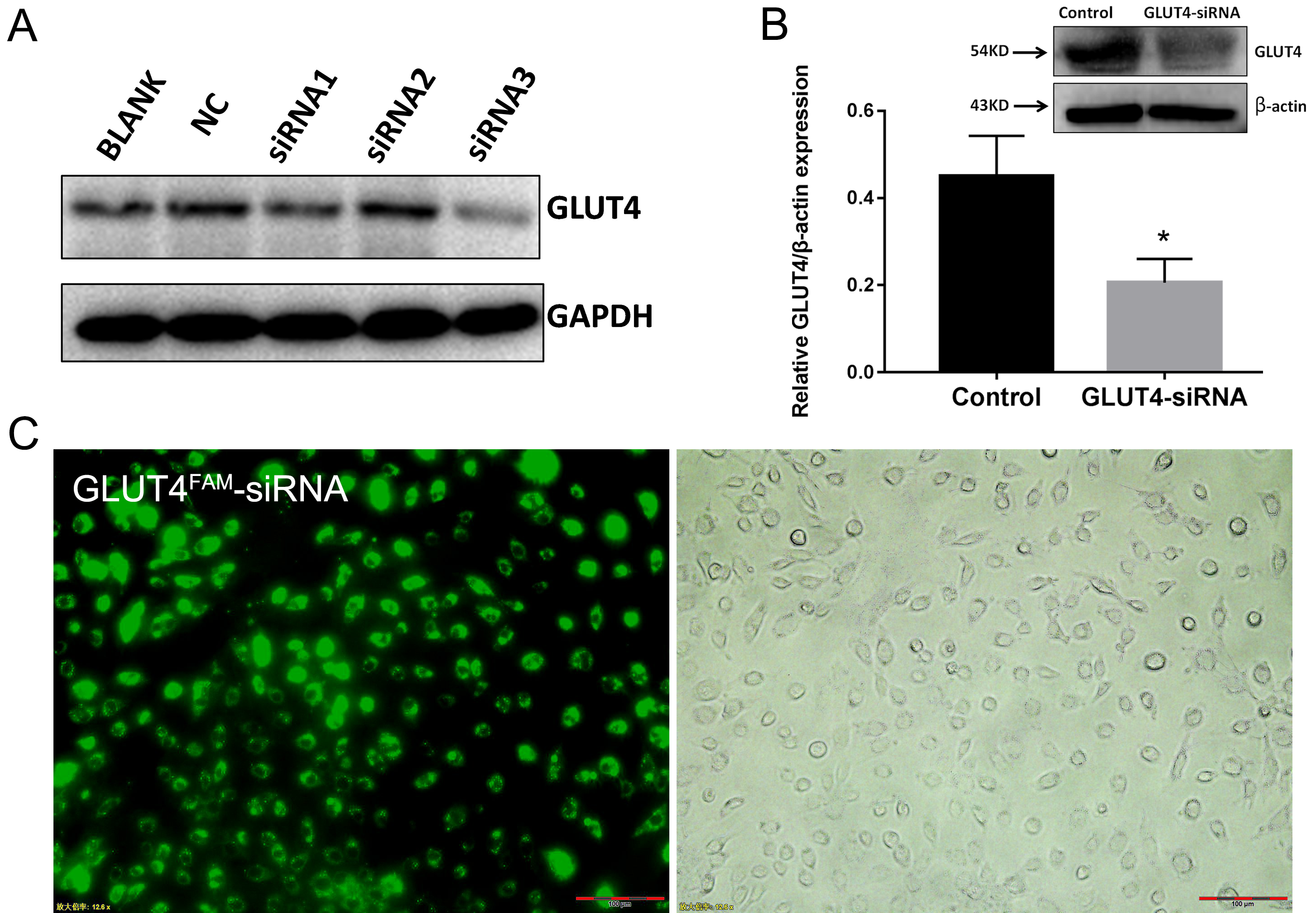

Supplement: Supplementary Figure 2 — In vitro interference efficiency of GLUT4-siRNA in mouse EECs. Three pairs of GLUT4 small interfering RNAs (siRNAs), were designed and transfected separately into Ishikawa cells, and the interference efficiency of each pair were determined by qPCR (data not shown) and western blotting at 72 h post-transfection. The siRNA pair with the strongest inhibitory effect on GLUT4 expression was modified with cholesterol, and then used in subsequent in vitro and in vivo experiments. (A) GLUT4 protein expression in Ishikawa cells was inhibited over 80% with Seq.3 siRNA compared with controls at 72 h. GAPDH served as loading control. (B) Western blotting for GLUT4 protein expression in mouse EECs following GLUT4-siRNA transfection. *P < 0.05 vs. Control. (C) The green FAM signals (>90%) in mouse EECs following GLUT4FAM-siRNA transfection. Scale bar, 500 μm. The experiment was repeated three times. [file Image_2.TIF]

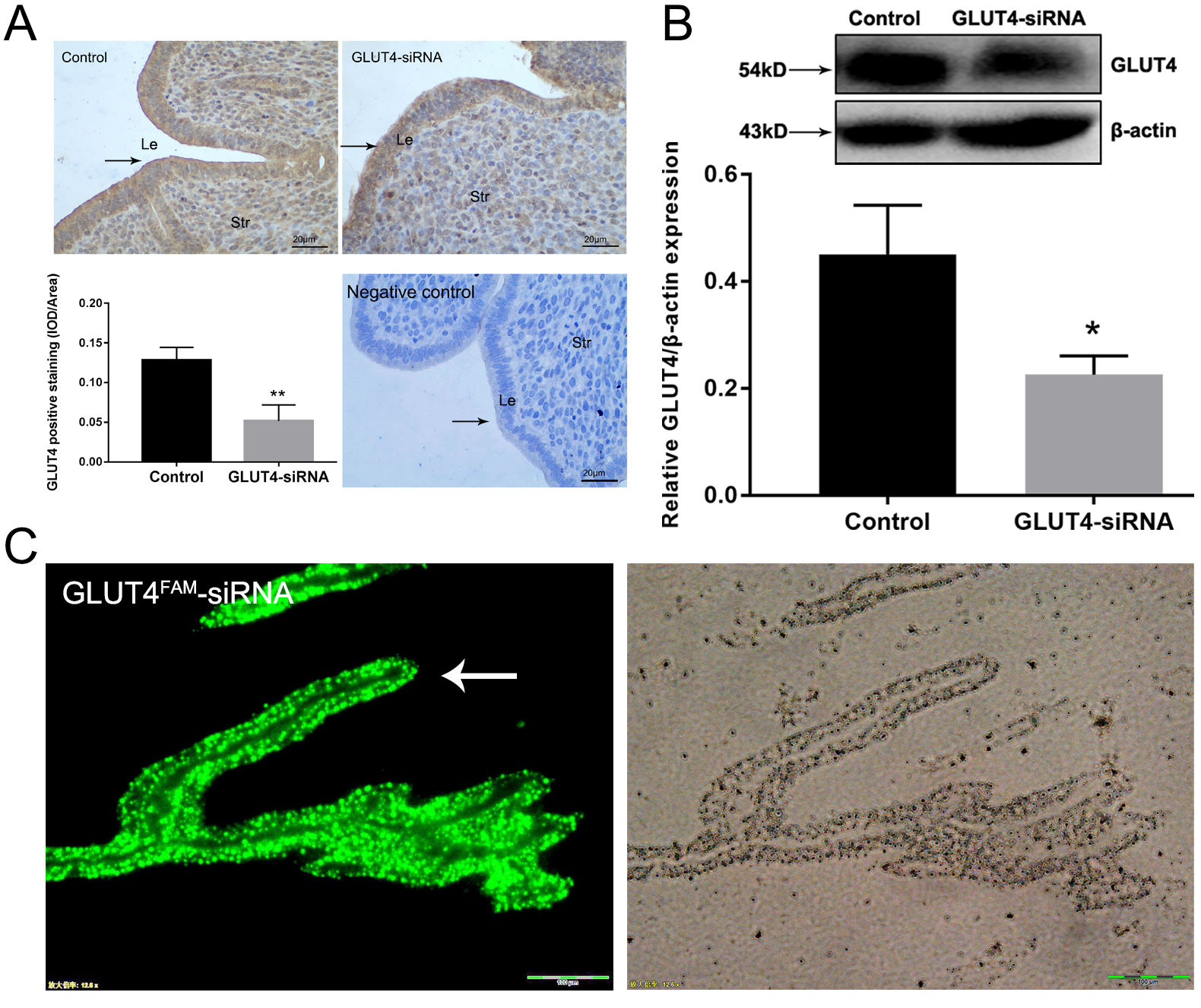

Supplement: Supplementary Figure 3 — In vivo efficiency interference of GLUT4-siRNA on pregnant day 4. (A) IHC for GLUT4 expression on pregnant day 4 following GLUT4-siRNA transfection. GLUT4 positive staining was analyzed by image Pro-Plus 6.0 software. Integrated optical density per area (IOD/Area). Negative control is without the primary antibody. Scale bars, 20 μm. (B) Western blotting for GLUT4 expression on pregnant day 4 following GLUT4-siRNA transfection. (C) The green signals confirmed that GLUT4FAM-siRNA was visibly transfect into the luminal epithelium on pregnant day 4. The white arrow indicates the luminal epithelium. Scale bars: 100 μm. *P < 0.05, **P < 0.01 vs. Control. The experiment was repeated three times. [file Image_3.TIF]

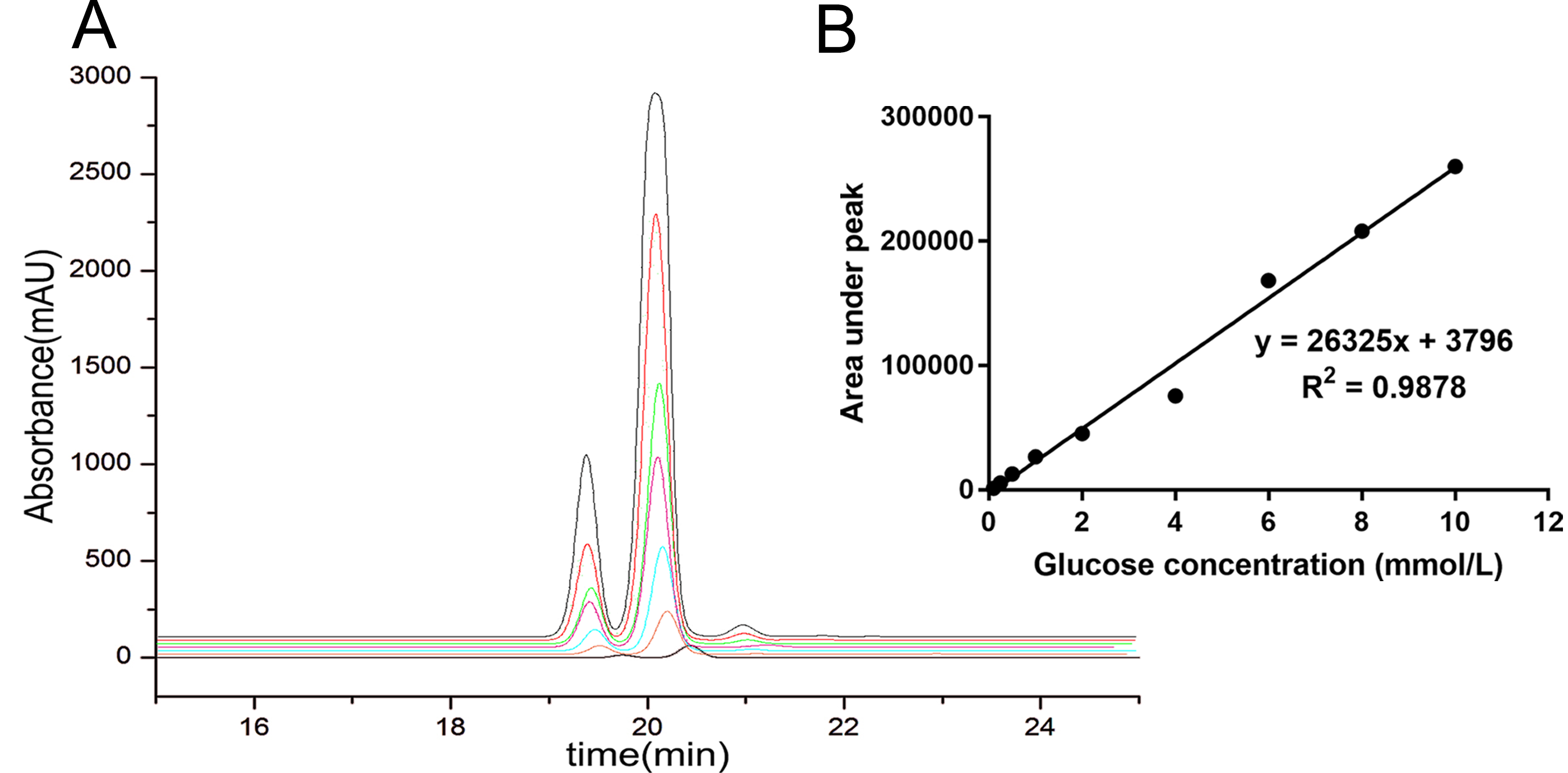

Supplement: Supplementary Figure 4 — Glucose concentration in uterine fluid detected by high performance liquid chromatography (HPLC). (A) Absorbance curve of different glucose standard solutions detected by HPLC. Characteristic peak of glucose in HPLC appeared at about 20 min. (B) Standard curve was established by glucose standard solutions with their HPLC absorbance values, and this equation was used to calculate glucose concentration in samples. [file Image_4.TIF]
